# Application and Mechanisms of Triptolide in the Treatment of Inflammatory Diseases—A Review

**DOI:** 10.3389/fphar.2019.01469

**Published:** 2019-12-06

**Authors:** Kai Yuan, Xiaohong Li, Qingyi Lu, Qingqing Zhu, Haixu Jiang, Ting Wang, Guangrui Huang, Anlong Xu

**Affiliations:** ^1^School of Life Sciences, Beijing University of Chinese Medicine, Beijing, China; ^2^Beijing Research Institute of Chinese Medicine, Beijing University of Chinese Medicine, Beijing, China; ^3^State Key Laboratory of Biocontrol, Department of Biochemistry, School of Life Sciences, Sun Yat-Sen (Zhongshan) University, Guangzhou, China

**Keywords:** triptolide, inflammatory disorders, anti-inflammation, immunosuppression, pubmed, Embase

## Abstract

Bioactive compounds from medicinal plants with anti-inflammatory and immunosuppressive effects have been emerging as important sources of drugs for the treatment of inflammatory disorders. Triptolide, a diterpene triepoxide, is a pharmacologically active compound isolated from *Tripterygium wilfordii* Hook F (TwHF) that is used as a remedy for inflammatory and autoimmune diseases. As the most promising bioactive compound obtained from TwHF, triptolide has attracted considerable interest recently, especially for its potent anti-inflammatory and immunosuppressive activities. Over the past few years, an increasing number of studies have been published emphasizing the value of triptolide in the treatment of diverse inflammatory disorders. Here, we systematically review the mechanism of action and the therapeutic properties of triptolide in various inflammatory diseases according to different systematic organs, including lupus nephritis, inflammatory bowel disease, asthma, and rheumatoid arthritis with pubmed and Embase. Based on this review, potential research strategies might contribute to the clinical application of triptolide in the future.

## Introduction

Complementary and alternative medications, including traditional Chinese medicines (TCMs), have long been used to treat inflammatory disorders, and they are generally well tolerated by patients (Goldrosen and Straus, 2004). The use of TCMs has necessitated urgent research into the mechanisms of action of natural products. One TCM, *Tripterygium wilfordii Hook F* (TwHF), has been used in folk medicine for the treatment of a variety of inflammatory disorders for many centuries (Tao et al., 1991; Goldbach-Mansky et al., 2009; Lv et al., 2015). TwHF belongs to celastraceae family and *Tripterygium* genus. It has been collected in Southern China and its roots have been used in various preparations to “relieve stasis and internal warmth,” among many other conditions diagnosed by TCM practitioners. TwHF was used to deal with rheumatoid arthritis and psoriasis in ancient China. In addition, TwHF was also used as a method of birth control in men. Previous studies demonstrated TwHF exhibited multiple pharmacological activities including antitumor, immune modulation, anti-inflammatory, and antifertility effects. Especially in RA, TwHF was found to have anti-inflammatory and cartilage protective effects (Zhou et al., 2018). However, TwHF might have significant side effects and severe toxicity, which limits the clinical application.

**Figure 1:**
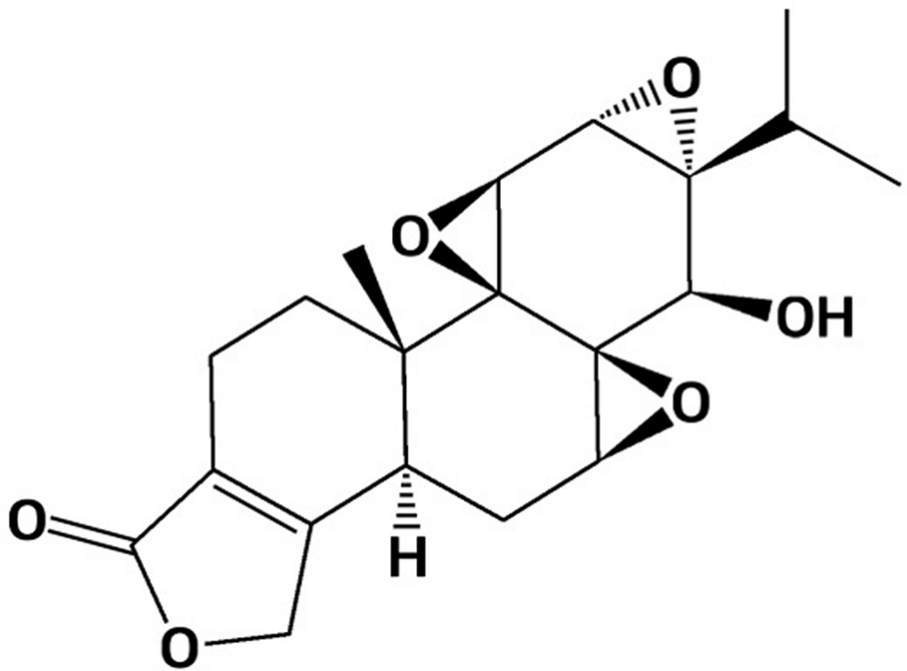
Chemical structure of triptolide.Triptolide has been shown to possess a broad spectrum of anti-inflammatory and immunosuppressive properties in treating various inflammatory disorder models. Based on potent anti-inflammatory biological activities, triptolide has increasingly drawn attention worldwide in recent decades.

Triptolide is a major bioactive compound derived from *T. wilfordii Hook F* (Kupchan et al., 1972). It is a diterpene triepoxide containing three epoxy groups, a C-14-hydroxyl group and a lactone ring (**Figure 1**).

With pubmed and Embase, we systematically review the therapeutic properties of triptolide in inflammatory diseases according to different systematic organs and illustrate its potential clinical applications.

## Potential Effects of Triptolide on Inflammatory Diseases

The therapeutic potential of triptolide has been tested in various inflammatory and autoimmune disorder models, including nephritis, asthma, arthritis, and neurodegenerative disorders, and triptolide has been found to modulate a wide variety of inflammatory mediators. These disorders and their inflammatory mediators will be discussed in brief below.

### Renal Diseases

#### Membranous Nephropathy

Membranous nephropathy (MN) is one of the major causes of nephrotic syndrome in adults and is characterized by subepithelial deposition of immune complexes (Cattran and Brenchley, 2017). Globally, the overall incidence of membranous nephropathy (MN) is estimated as 1/100,000. Immune-mediated podocyte injury is considered to underlie the proteinuria in MN. Asymptomatic proteinuria and generalized edema are clinical presentations of MN. Researchers found that triptolide could reduce podocyte injuries in MN to reduce proteinuria and alleviate inflammatory response in animal model of MN.

Chen et al. (2010) demonstrated that 200 µg/kg/day triptolide could effectively reduce proteinuria and inhibit immune-mediated injuries in an experimental rat model of MN. The recovery of podocyte injuries was promoted after triptolide treatment, accompanied by a reduction in glomerular complement component 5b-9 (C5b-9) deposits. In addition, Triptolide also suppressed reactive oxygen species (ROS) generation and p38 mitogen-activated protein kinase (MAPK) activation in the podocytes induced by C5b-9.

Later, Zhou et al. (2016) showed that 200 µg/kg/day triptolide attenuated the inflammatory response in MN rats *via* suppression of the nuclear factor-kappa B (NF-κB) signaling pathway. Interestingly, they also found that triptolide treatment could significantly decrease malondialdehyde (MDA) levels while enhancing superoxide dismutase (SOD) activity in the serum to reduce oxidative stress and inflammatory responses.

More recently, Chen et al. (2017) revealed that 100 and 200 µg/kg/day triptolide reduces podocyte injury by inhibiting podocyte apoptosis in an experimental rat model of MN. Cleaved caspase-3 and cleaved poly ADP-ribose polymerase (PARP) were markedly decreased after triptolide treatment. Triptolide inhibited C5b-9-induced MAPK activation in podocytes by decreasing p-JNK and p-ERK expression.

So, triptolide could alleviate membranous nephropathy by inhibiting inflammatory signaling pathways including NF-κB and MAPK pathways. Oxidative stress and apoptosis were also involved in the mechanism of triptolide against MN. However, the researchers have not investigated the triptolide cytotoxicity in the organ of animal model of MN. Only Chen et al. (2017) studied aminotransferase in the serum.

#### Lupus Nephritis

Lupus nephritis is inflammation of the kidney caused by systemic lupus erythematosus and is characterized by increased production of cytokines and autoantibodies, deposition of immune complexes, and infiltration of leukocytes (Lech and Anders, 2013). Lupus nephritis (LN) influences almost 40% of patients with systemic lupus erythematous (SLE).

Tao et al. (2008) discovered that triptolide with 15 weeks administration could increase the survival rate, reduce disease severity and decrease cytokine production in mice with lupus nephritis (LN). They indicated that the survival rate of mice was significantly higher in the triptolide group (87.5%) than in the vehicle group (35.7%). Triptolide could decrease proteinuria and blood urea nitrogen levels were significantly reduced in the triptolide-treated mice compared with the control mice throughout the treatment period. Histopathologic analysis showed that triptolide-treated mice had less severe kidney disease, with significantly diminished glomerular and interstitial disease. In this study, the NZB/NZWF1 mice were used as the animal model of LN. There are some shortcomings of NZB/NZWF1 mice. These mice do not possess some clinical manifestations of lupus such as arthritis and rash. Another drawback of this strain is the long disease incubation time almost 6 months which is the long disease incubation time.

#### Kidney Transplantation

Due to allograft rejection, the fate of long-term grafts has not changed significantly over the past decades. Allograft rejection, characterized by the activation of various inflammatory cells, cytokines, chemokines, and adhesion molecules, is one of the leading causes of graft loss in clinical transplantation (Ingulli, 2010). Triptolide could prolong the survival of kidney transplantation by inhibiting inflammatory activities.

In 2009, an experimental study demonstrated that triptolide with 250 and 500 µg/kg/day treatment effectively prolongs allograft survival (Zhang et al., 2009). Brown Norway rat kidneys were transplanted into Lewis recipients to generate allograft groups, and graft recipients were treated with 0.25 and 0.5 mg/kg/day triptolide for 14 days. The average median survival time (MST) was 18 and 19.8 days in the 0.25 and 0.5 mg/kg/day triptolide treatment groups, respectively. There had some flaws in this study. The researchers have not evaluated the cytotoxicity of triptolide in the healthy organs of kidney transplantation animal model.

Later, Zhang et al. (2013) demonstrated that complex phenotypic and allostimulatory functional changes induced in rat bone marrow-derived dendritic cells (DCs) by 1-10 nM triptolide may account for the prolonged allograft survival. They also found that triptolide-conditioned DCs could induce allospecific T-cell regulation and prolong renal graft survival.

Crews et al. (2005) demonstrated that downregulation of vascular cell adhesion molecule 1 (VCAM-1) and transforming growth factor beta (TGF-β) are associated with successful 500 µg/kg/day triptolide with 10 days treatment of chronic allograft rejection in rats. The gene expression levels of VCAM-1 and TGF-β exhibited significant associations with the CADI scores of the experimental groups.

Importantly, Hong et al. (2002) showed triptolide is a potent suppressant of C3, CD40 and B7h expression in activated human proximal tubular epithelial cells (PTECs). Triptolide exerts inhibitory effects on C3, CD40, and B7h expression in TNF-α-stimulated PTECs.

Therefore, triptolide might have a beneficial effect on kidney transplantation by inhibiting inflammatory molecules such as VCAM-1, TGF-β, C3, and CD40.

#### Renal Fibrosis

Renal fibrosis is associated with a decline in renal excretory function, and unresolved inflammation promotes progressive renal fibrosis, which can culminate in end-stage renal disease (Zhou et al., 2016). It has been found that 0.6 mg/kg per day triptolide attenuates renal interstitial fibrosis by decreasing α-SMA and TGF-β1 expression and interstitial collagen deposition in the kidney (Yuan et al., 2011). Triptolide could also inhibit macrophage and myofibroblast infiltration.

### Gastrointestinal Disease

#### Inflammatory Bowel Disease

Inflammatory bowel disease (IBD) includes ulcerative colitis and Crohn’s disease (CD) and is characterized by chronic inflammation of the gastrointestinal tract (Abraham and Cho, 2009). An estimated 1.6 million Americans are affected by IBD, and approximately 2.5–3 million people in Europe currently have IBD. Previous studies found that triptolide could deal with IBD in mouse model and patients.

Two kinds of inflammatory bowel disease animal models including dextran sulfate sodium (DSS) induced colitis mice and interleukin-10 gene-deficient (IL-10^−/−^) mice were adopt in these studies. 0.07 mg/kg/day Triptolide with 8 weeks treatment alleviated diarrhea, edema, hyperemia, and inflammatory cells infiltration in the animal models (Wei et al., 2008). In addition, researchers also investigated the mechanism of triptolide in the treatment of IBD. Li et al. (2010) found that triptolide ameliorates colitis by suppressing the IL-6/signal transducer and activator of transcription 3 (STAT3) signaling pathway and downregulating IL-17. Zhang et al. (2014) found that 0.6 mg/kg/day triptolide administration ameliorates intestinal injury by inhibiting the expression of secondary lymphoid tissue chemokine chemokine (C-C motif) ligand 21 (CCL21), which has chemotactic effects on T cells, B cells, NK cells, and DCs. However, they only investigated the expression of CCL21, which is not sufficient to demonstrate triptolide influence the chemotactic effects of T cells, B cells, NK cells, and DCs.

Inflammatory bowel disease is associated with an increased risk of developing colorectal cancer. Wang et al. demonstrated that triptolide with 0.1, 0.3, or 1 mg/kg/day for 20 weeks inhibited colitis-related colon cancer progression *in vivo via* downregulating Rac1 and the Janus kinase (JAK)/STAT3 pathway (Wang et al., 2009). *In vitro* cell cycle analysis revealed that triptolide inhibits the proliferation, migration and colony formation of colon cancer cells. Triptolide could reduce the secretion of IL6 and levels of JAK1 and IL6R by interrupting the IL6R-JAK/STAT pathway. The shortcoming in this study was that the high dose group was 1 mg/kg/day which might lead to organ damage. However, Wang et al. have not investigated the organ damage in this group.

#### Intestinal Fibrosis

Intestinal fibrosis is a common complication of inflammatory bowel disease that is characterized by abnormal deposition of extracellular matrix proteins produced by activated myofibroblasts in the intestine (Rieder and Fiocchi, 2008). Tao et al. (2015) discovered that 45 mg/kg per day triptolide ameliorates colonic fibrosis in an experimental rat model. Triptolide could reduce collagen production and extracellular matrix deposition in the colon. Collagen I protein and collagen Iα1 transcript expression were also inhibited after treatment in the isolated subepithelial myofibroblasts of rats with colonic fibrosis.

#### Liver Fibrosis

Liver fibrosis is the excessive accumulation of extracellular matrix that occurs in most types of chronic liver diseases (Bataller and Brenner, 2005). Chong et al. (2011) found that 20 µg/kg triptolide exerts antihepatofibrotic effects in animal model of liver fibrosis. Triptolide inhibited the NF-κB signaling pathway in hepatic stellate cells. In addition, triptolide treatment reduced hepatic fibrosis scores *in vivo*.

### Respiratory Disease

#### Asthma

Asthma is a common long-term inflammatory airway disorder. Airway smooth muscle cells, goblet cells and eosinophils contribute to asthmatic airway inflammation (Cohn et al., 2004). Approximately 8% of the adult population in worldwide are diagnosed asthma. Triptolide could inhibit asthma airway remodeling by suppressing the inflammatory signaling pathway, interfering with the production of pro-inflammatory chemokines and cytokines.

In the ovalbumin (OVA)—sensitized asthma mice model, Chen et al. showed that 40 µg/kg/day triptolide treatment may function as an inhibitor of asthma airway remodeling (Chen et al., 2011; Chen et al., 2015b).Triptolide could inhibit mucous gland hypertrophy, goblet cell hyperplasia and collagen deposition through the suppression of TGF-β1/Smad and NF-κB signaling pathways in airways. However, they only tested p-P65 protein in NF-κB signaling pathway which was not sufficient to demonstrated NF-κB signaling pathway was involved in the mechanism of triptolide. Furthermore, they revealed that triptolide inhibits the proliferation and migration of rat ASMCs (Chen et al., 2015a). Triptolide exerts a time- and dose-dependent inhibition of TGF-β1-induced ASMC proliferation by blocking S and G2/M phases without apparent cytotoxic effects.

Apart from signaling pathways, researchers also focused on the inflammatory cells in asthma. Ji et al. (2015) have shown that triptolide modulates the CD4^+^ T cell balance in an asthmatic animal model. Triptolide could regulate Th1/Th2 balance and Th17/Treg equilibrium. Inflammatory cytokines in the bronchoalveolar lavage fluid (BALF), such as IL-10, IL-13, IL-17, TNF-α, and TGF-β, are downregulated after treatment. Mao et al. (2008) found that 2 weeks of 40 µg/kg/day triptolide treatment alleviated eosinophil recruitment in BALF by inhibiting bone marrow eosinophilopoiesis. The number of eosinophils in the peripheral blood and bone marrow were significantly reduced after triptolide treatment

#### Acute Lung Injury

Acute lung injury (ALI) is a disorder of acute inflammation consisting of acute hypoxemic respiratory failure (Rubenfeld et al., 2005). Triptolide was found to alleviate ALI in LPS-induced mouse model and chlorine exposure mouse model.

In mouse models of chlorine gas-induced acute lung injury, triptolide ranged 100–1,000 µg/kg/day administration showed anti-inflammatory effects by decreasing neutrophils infiltration to the lung lavage fluid and lung tissue (Hoyle et al., 2010). In the LPS-induced ALI mouse model, 1–50 µg/kg/day triptolide ameliorated ALI by inhibiting the NF-κB signaling pathway (Wang et al., 2014). Triptolide inhibited chemokines such as macrophage inflammatory protein alpha (MIP-1α), MIP-1β, regulated upon activation normal T cell express sequence (RANTES) in the lung tissue and inflammatory cytokines such as TNF-α, IL-1β, IL-6 in the BALF of mice with ALI. Suppressed expression of p-IκB-α and p-NF-κB p65 showed that triptolide inhibits the NF-κB signaling pathway. Moreover, in the same animal model, Wei et al. found that triptolide with 5, 10, and 15 µg/kg treatment attenuated the LPS-induced inflammatory response by inhibiting the MAPK signaling pathway (Wei and Huang, 2014). Triptolide inhibits the phosphorylation of p38, JNK, and ERK. However, there have some significant disadvantages in LPS-induced ALI mouse model. LPS preparations might be contaminated with bacterial materials and other bacterial lipoproteins. LPS does not induce injury of epithelial and endothelial cells occurring in acute respiratory distress syndrome of humans.

#### Pulmonary Arterial Hypertension

Pulmonary arterial hypertension (PAH) is an incurable disease characterized by increased blood pressure in the arteries of the lungs (Farber and Loscalzo, 2004). There is an increasing appreciation of inflammation in the pathogenesis of PAH with an accumulation of inflammatory cells and elevated cytokines. Triptolide could attenuate the development of pulmonary hypertension by down-regulating expression of functionally related genes.

In a rat experimental pulmonary arterial hypertension model, it has been shown that 0.25 mg/kg/day triptolide can effectively attenuate the development of pulmonary hypertension (Faul et al., 2000). By Day 35, the mean pulmonary arterial pressure was diminished in triptolide-treated rats compared with vehicle-treated rats. Triptolide-treated rats also showed significantly less pulmonary arterial neointimal formation and right ventricular hypertrophy. Another study analyzed longitudinal transcriptional expression in pulmonary hypertension rats administered 0.25 mg/kg/day triptolide treatment for 30 days (Vaszar et al., 2004). Transcriptional analysis with total lung RNA was performed for every experimental time point. They found that a group of functionally related genes, such as matrix metalloproteinase (MMP) and mast cell chymases, were significantly coexpressed with the development of PAH. The global gene expression pattern also resembled that seen in intermediate stages of severity. Functionally related genes were downregulated in response to triptolide treatment. Monocrotaline (MCT)-induced pulmonary hypertension (MCTP) was used as animal model in these two studies. Compared with chronic hypoxia PAH animal model, MCTP is easily to be therapeutically improved owning to the acute nature, which is not alike the characteristics of PAH in human.

#### Pulmonary Fibrosis

Pulmonary fibrosis is a chronic, debilitating and lethal lung disorder. Pulmonary fibrosis is estimated to have prevalence of 13 to 20 per 100,000 people worldwide. It has been demonstrated that 0.25 mg/kg triptolide effectively reduced radiation-induced lung fibrosis (Yang et al., 2015; Chen et al., 2016). Triptolide improved the pulmonary function by inhibiting myofibroblast activation, collagen deposition and ROS production in lung tissues. Triptolide also mitigated pulmonary fibrosis partly by downregulating nicotinamide adenine dinucleotide phosphate-oxidase 2 (NOX2) through the NF-κB pathway.

### Endocrine Diseases

#### Diabetic Nephropathy

Diabetic nephropathy (DN) is a serious complication in those with diabetes mellitus. Podocyte injuries, such as decreased density of podocytes due to chronic inflammation and oxidative stress, are observed in the development of diabetic glomerular injury (Navarro-González and Mora-Fernández, 2008). Triptolide exerted protective effects on DN of DN animal model.

In an *in vitro* model of db/db diabetic mice with increased albuminuria, it has been revealed that triptolide markedly attenuates albuminuria. It has been shown that 50 µg/kg/day triptolide with 12 weeks treatment attenuates inflammation in the kidneys accompanied by alleviated podocyte injury. Triptolide could reduce the expression of desmin protein, MCP-1 protein and CD68-positive macrophages in db/db diabetic mouse kidneys (Gao et al., 2010).

Apart from db/db diabetic animal model, investigators also used streptozocin-induced DN model to reveal the mechanism of triptolide against DN. db/db diabetic animal model was used to be type 2 diabetes model, while streptozocin-induced DN model was used to be type 1 diabetes model.

Ma et al. demonstrated that 8 weeks of 100 µg/kg/day triptolide administration significantly reduces the expression of TGF-β1 and osteopontin to alleviate inflammation in the kidney. (Ma et al., 2013). Later, Guo et al. discovered that 4 weeks of 6, 12, or 24 mg/kg/day triptolide improves DN by regulating Th cell balance and macrophage infiltration (Guo et al., 2016). Triptolide could inhibit proinflammatory cytokines and increase anti-inflammatory cytokines by regulating the Th1/Th2 cell balance. Triptolide also inhibits macrophage infiltration and macrophage-mediated inflammation in the kidneys. Han et al. (2017) confirmed that triptolide significantly inhibits mesangial cell proliferation by suppressing the PDK1/Akt/mTOR pathway. More recently, Dong et al. (2017) reported that triptolide alleviates oxidative stress in the kidneys of DN. Renal homogenate SOD and MDA were significantly downregulated after treatment.

So, Th cell balance, macrophage, oxidative stress might be involved in the mechanism of triptolide in treating DN.

#### Diabetic Cardiomyopathy

Diabetic cardiomyopathy is one of the leading cardiovascular complications in diabetic patients. Chronic inflammation plays an important role in diabetic cardiomyopathy (Frati et al., 2017).

Studies have suggested that triptolide ranged 100, 200, or 400 µg/kg/day improves cardiac diastolic and systolic function in diabetic rats (Wen et al., 2013; Guo et al., 2016). Triptolide treatment prevents myocardial fibrosis and collagen accumulation in diabetic myocardium by decreasing the expression of cardiac inflammatory mediators including TGF-β1, α-SMA, TNF-α, IL-1β, and vimentin. Triptolide treatment also inhibits the recruitment of macrophages and T lymphocytes in diabetic rat hearts. The inhibitory effect of triptolide on diabetic cardiomyopathy might be mediated by the suppression of the NF-κB immune pathway. More recently, Liang et al. (2015) detected that 100, 200, or 400 µg/kg/day triptolide improves cardiac function and increases cardiac energy metabolism by activating the MAPK signaling pathway.

Thus, triptolide could inhibit inflammatory cells recruitment and cytokines expression to reduce myocardial fibrosis, apoptosis and necrosis in diabetic cardiomyopathy. The shortcomings of these studies were that the researchers only tested N-κB p65 in NF-κB signaling pathway and p38 MAPK protein in MAPK signaling pathway when they studied the related pathways. Only one protein in the inflammatory signaling pathway was not persuasive to demonstrate the related pathways were involved in the mechanism.

### Rheumatic Diseases

#### Rheumatoid Arthritis

**Figure 2:**
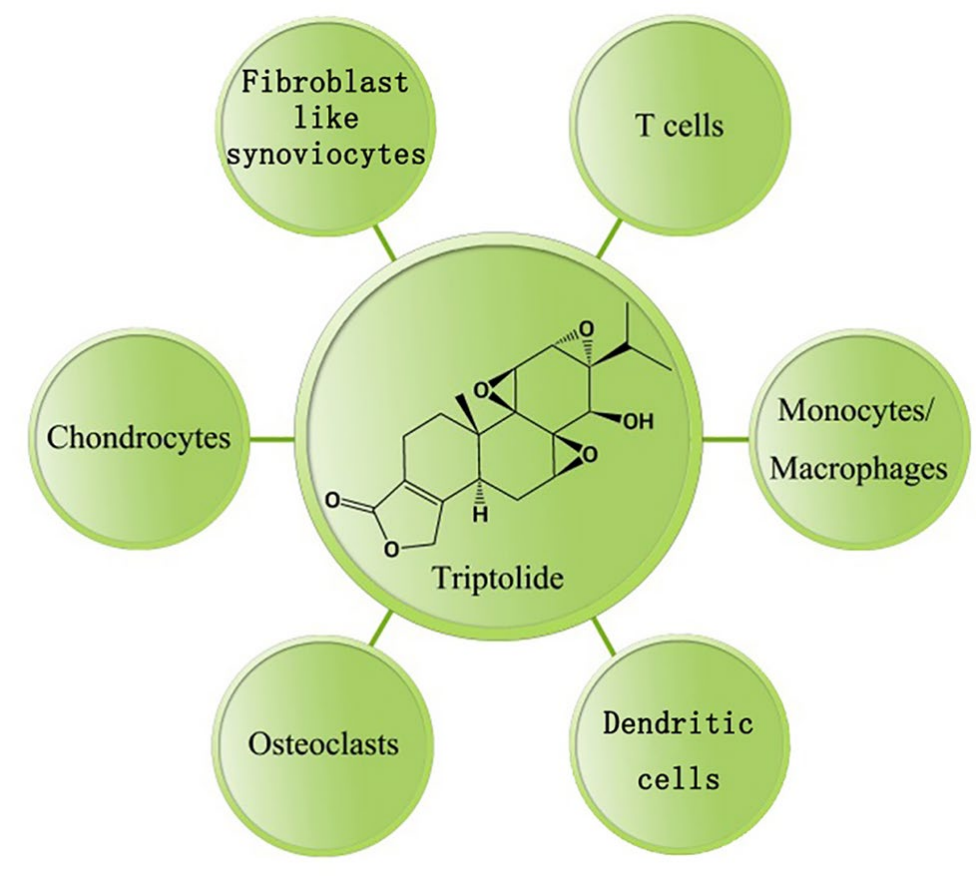
Anti-inflammatory effect(s) of triptolide on RA.Triptolide has a broad array of cellular mechanisms and potential therapeutic applications (**Table 1**).

Rheumatoid arthritis (RA) is a systemic inflammatory autoimmune disorder in which genetic and environmental risk factors contribute to disease development (Collison, 2016). Descriptive epidemiology studies of RA showed a population prevalence of 0.5–1% in worldwide. The disease is characterized by inflammation of synovial joints leading to the destruction of articular cartilage and erosion of the bone. Fibroblast-like synoviocytes (FLS), T cells, macrophages, DCs, osteoclasts, and chondrocytes play important roles in the pathogenesis of RA. Previous studies have observed antirheumatic properties of triptolide. In an animal model of RA, triptolide ameliorates the severity of arthritis (Gu and Brandwein, 1998). The anti-inflammatory effects of triptolide in dealing with RA have thus far been attributed to the aspects described in **Figure 2**.

**Table 1:** Cellular target(s) modulated by triptolide in rheumatoid arthritis.

Type of cells	Mechanism(s) of action
Fibroblast-like synoviocytes	Inhibits viability, proliferation, migration and invasive capacities. Affects cytoskeletal rearrangement. Arrests the cell cycle. Induces apoptosis. Reduces inflammatory mediators, including IL-18, prostaglandin E2 (PGE-2), cyclooxygenase-2 (COX-2), pro-MMP1 and 3. Inhibits the NF-κB and MAPK pathways.
T cells	Inhibits proliferation and differentiation. Induces apoptosis. Reduces inflammatory cytokines, including IL-2, IL-13, IL-17 and IFN-γ. Inhibits the NF-κB and MAPK pathways.
Monocytes/ Macrophages	Reduces inflammatory cytokines, including IL-1β, IL-6, IL-8, IL-12, IL-37, TNF-α and IFN-γ. Reduces inflammatory chemokines, including CCL-1, 2, 5, 7, and 12 as well as chemokine (C-X-C motif) ligand (CXCL)-10 and 11. Reduces the expression of costimulatory molecules CD80 and CD86. Reduces production of nitric oxide (NO), superoxide anion and ROS. Induces apoptosis. Inhibits the NF-κB pathway and TAK1 kinase activity.
Dendritic cells	Inhibits differentiation, maturation, allostimulatory capacities, chemotactic responses and migration. Induces apoptosis. Reduces inflammatory mediators, including MIP-1α, MIP-1β, MCP-1, PGE-2 and COX-2. Reduces the expression of costimulatory molecules CD1a, CD40, CD80 and CD86. Inhibits the PI3-K/Akt and NF-κB pathways.
Osteoclasts	Inhibits differentiation and osteoclastogenesis. Regulates the RANKL/RANK/osteoprotegerin signaling pathway. Inhibits NF-κB activation.
Chondrocytes	Reduces inflammatory mediators, including TNF-α, IL-6, PGE-2, COX-2, MMP-3 and MMP-13. Inhibits aggrecanase-1 expression. Inhibits NF-κB activation.

##### Fibroblast-Like Synoviocytes

Fibroblast-like synoviocytes, also known as synovial fibroblasts, are special cells that play a crucial role in the pathogenesis of RA (Bartok and Firestein, 2010). In RA, FLSs are the most common cell type at the cartilage–pannus junction and perpetrate inflammation through their massive production of inflammatory cytokines, chemokines, and matrix-degrading molecules and through immigration and invasion of joint cartilage (Bustamante et al., 2017).

Triptolide has been found to inhibit inflammation of fibroblast-like synoviocytes in several studies. Triptolide with 100 ng/ml concentration could inhibit inflammatory cytokine production of FLS and inhibit invasive capacities of FLS. Lu et al. (2008) demonstrated that triptolide inhibits the expression of IL-18 and its receptor in PMA-stimulated FLSs in a dose-dependent manner by suppressing NF-κB activity. However, Lu et al. (2008) only analyzed one inflammatory cytokine produced by FLS. Lin et al. (2001) demonstrated that 28–140 nM triptolide suppresses the production of proMMP1 and 3 in IL-1α-induced FLSs. In addition, triptolide downregulates the expression of PGE-2 in activated FLSs by selectively suppressing the production of COX-2. Kusunoki et al. (2004) revealed that 100nM triptolide decreases viability, inhibits proliferation, and induces apoptosis of FLSs in a concentration-dependent manner at very low concentrations. Later, Yang et al. (2016) discovered that 10–50 nM triptolide reduces the migratory and invasive capacities of FLSs by affecting the cytoskeletal rearrangement of FLSs. They also indicated that triptolide blocks the activation of the JNK MAPK pathway in FLSs. Thus, triptolide suppresses the inflammatory mediators including IL-18, COX-2, proMMP1 and 3 in activated FLS. NF-κB and MAPK pathways might be involved in the mechanism of inhibiting FLS inflammatory activities.

##### T Cells

T cells take center stage in the pathogenesis of rheumatoid arthritis (Cope et al., 2007). The predominance of T cells in lymphocytic infiltrates in the tissue of patients with RA has been defined.

Previous studies have demonstrated the immunosuppressive effects of triptolide in regulating T cell function. Inflammatory cytokine production, apoptosis, cell proliferation, and inflammatory pathways were involved in the effects of 10–500 nmol triptolide on T cells. Inflammatory cytokines production of T cells including IL-2, IFN-γ, IL-13, IL-17 were inhibited by triptolide (Chan et al., 1999; Dai et al., 2013). Qiu et al. (1999) reported that triptolide inhibits IL-2 expression in T cells at the level of purine-box/nuclear factor of activated T cells and NF-κB transcriptional activation triggered by all stimuli examined. Dai et al. (2013) demonstrated that triptolide inhibits IL-13 expression by inhibiting GATA3 and nuclear factor of activated T cells 1 (NFAT1) nuclear translocation and their binding rates to the IL-13 gene promoter region. However, Dai et al. have not revealed the relationship between IL-13 and GATA3 in T cells clearly. They only examined the expression of IL-13 and GATA3 after triptolide administration.

In the aspects of apoptosis and signaling pathways, Yang et al. (1998) discovered that 10–100 ng/ml triptolide induces T cell apoptosis accompanied by increased caspase activity and degradation of the caspase substrate PARP. Liu et al. (2000) showed that 2–10 µg/L triptolide attenuates T cell NF-κB activation by upregulating IκBα mRNA expression. In addition, Ho et al. (2013) discovered that 50–200 ng/ml triptolide attenuates both the NF-κB and MAPK signaling pathways.

##### Monocytes/Macrophages

Monocytes/macrophages play an important role in the pathogenesis of rheumatoid arthritis (Davignon et al., 2013). A number of monocytes/macrophages infiltrate into the rheumatoid synovium. They are a potent source of cytokines, chemokines and ROS that participate in the initiation, maintenance, and resolution of inflammation. The number of macrophages in the inflamed synovial membrane and cartilage-pannus junction are correlated with cartilage and bone destruction in RA (Kinne et al., 2007). As such, monocytes and macrophages are viewed as relevant therapeutic targets in treating RA.

Triptolide has been found to inhibit inflammation of monocytes and macrophages. Triptolide inhibited inflammatory cytokines and costimulatory molecules in monocytes. Triptolide suppressed the production of IL-12 in the THP-1 human monocytic leukemia cell line at dosage of 2.5–0.625 µg/L (Liu et al., 2005). Triptolide also suppressed expression of the costimulatory molecules CD80 and CD86 on LPS-activated THP-1 cells. In addition, 5–25 nM triptolide prompted THP-1 apoptosis by inhibiting the NF-κB pathway and activating the MAPK pathway (Park and Kim, 2013). Interestingly, MAPK pathway was activated in this study, which is different with other studies. Activated MAPK pathway might contribute to the apoptosis of monocytes induced by triptolide.

Apart from monocytes, triptolide inhibits the inflammatory activities of macrophages through multiple mechanisms. First, 5–40 ng/ml triptolide inhibits some key inflammation-related cytokines in LPS-activated macrophages, including TNF-α, IL-1β, IL-6, IL-8, and IFN-γ (Wu et al., 2006; Yang et al., 2010). Some proinflammatory chemokines, including CCL-1, 2, 5, 7, and 12, as well as CXCL-10 and 11, are attenuated after triptolide administration with concentrations as low as 10–50 nM (Matta et al., 2009). Second, several studies have revealed that 5–25 ng/ml triptolide suppresses nitric oxide (NO) and ROS production in activated macrophages (Wang et al., 2004; Bao et al., 2007). Kim et al. (2004) discovered that triptolide abrogates inducible NO synthase (iNOS) gene expression and inhibits NO production in a dose-dependent manner. Wu et al. (2006) found that triptolide inhibits superoxide anion and ROS production in murine peritoneal macrophages. Third, triptolide down-regulates NF-κB activation and JNK phosphorylation. Kim et al. (2004) revealed that triptolide significantly inhibits the DNA binding activity of NF-κB, whereas Premkumar et al. (2010) showed that myeloid differentiation primary response 88 (MyD88)-dependent and independent pathways of toll-like receptors (TLRs) are engaged in the biological activity of triptolide. So, triptolide could influence the inflammatory cytokines production, oxidative stress, and related inflammatory signaling pathways in the macrophages to alleviate inflammation.

##### Dendritic Cells

Dendritic cells play important roles in the induction of immunity and in mediating immune tolerance as professional antigen-presenting cells (Khan et al., 2009). In the synovial fluid of RA joints, DCs amplify immune responses by ingesting and presenting antigens to T cells (Bell et al., 2017).

It has been found that triptolide could suppress the migration and differentiation of dendritic cells DCs. Chen et al. (2005) found that triptolide is a potent suppressor of DCs maturation and trafficking. The allostimulatory capacities and chemotactic responses of DCs are inhibited by 2.5–10 nM triptolide over a pharmacologic concentration range. Zhu et al. (2005) discovered that 20 ng/ml triptolide prevents the differentiation of immature DCs by inhibiting CD1a, CD40, CD80, CD86 and HLA-DR expression. Thus, triptolide could inhibit the trafficking, maturation, and differentiation of DCs and. In another article, triptolide inhibits DC-mediated chemoattraction of neutrophils and T cells by inhibiting DC production of CC and CXC chemokines, including MIP-1α, MIP-1β, and MCP-1, in response to LPS (Liu et al., 2006). However, they have not revealed the mechanisms of triptolide inhibiting DC-mediated chemoattraction of other inflammatory cells. Later, Liu et al. (2007) revealed that 10–100 ng/ml triptolide impairs DC migration by inhibiting the phosphatidylinositol-3 kinase (PI3-K)/Akt and NF-κB pathways. Triptolide-mediated suppression of the NF-κB pathway, STAT3 phosphorylation and an increase in suppressor of cytokine signaling 1 (SOCS1) expression in DCs may be involved in the inhibitory effect of triptolide. (Liu et al., 2004).

##### Osteoclasts

Extensive bone destruction is a feature of patients with rheumatoid arthritis, leading to severe deformity of the affected joints. As a result, ameliorating bone destruction is a very important issue in the treatment of RA.

Triptolide has been shown to efficiently ameliorate the progression of bone destruction in rheumatoid arthritis by inhibiting osteoclast activities. The related signaling pathways might include receptor activator of the nuclear factor kappa-B ligand (RANKL)/RANK/osteoprotegerin (OPG) and NF-κB signaling pathways. Liu et al. (2013) showed that 8-32µg/kg/day triptolide prevents bone destruction and inhibits osteoclast formation in an animal model of RA by regulating the RANKL/RANK/OPG signaling pathway. Later, Huang et al. (2015) reported that 1.25nM triptolide inhibits osteoclastogenesis and osteoclastic bone resorption by suppressing RANKL-induced activation of NF-κB and p65 nuclear translocation in osteoclast-like cells. More recently, Xu et al. (2016) found that triptolide inhibits osteoclast differentiation and bone resorption by increasing expression of the immunosuppressive cytokines IL-10 and TGF-β1 by Tregs. Therefore, these data show that triptolide is effective in treating bone destruction by regulating osteoclast inflammatory activities, which is a hallmark of RA physiopathology.

##### Chondrocytes

Rheumatoid arthritis is characterized by synovitis in joints and destruction of cartilage. Cartilage is destroyed by enzymatic and mechanical processes. The chondrocytes themselves also synthesize cytokines and MMPs or respond to local cytokine release to accelerate articular cartilage destruction (Otero and Goldring, 2007).

Triptolide could reduce the expression of inflammatory mediators including TNF-α, IL-6, COX-2, and MMPs in chondrocytes to prevent cartilage damage. In 2005, Liacini et al. (2005) showed that triptolide at 125–250 nM concentrations possesses cartilage-protective effects by suppressing MMP expression in chondrocytes *in vitro*. 100nM triptolide inhibits proinflammatory cytokine-induced MMP-3 and MMP-13 expression in chondrocytes in a dose-dependent manner. Later, Lin et al. (2007) discovered that triptolide suppresses inflammation and cartilage destruction in RA mice. In 2009, Xiao et al. (2009) demonstrated that triptolide reduces the expression of the proinflammatory cytokines COX-2 and NF-κB in paw cartilage in arthritic rats. Immunohistochemistry staining revealed that triptolide inhibits the expression of TNF-α, IL-6, COX-2 and NF-κB in superficial cartilage. The flaw of this study was that they only test NF-κB protein in the tissue. Other proteins in the NF-κB signaling pathway have not been investigated.

### Neurologic Diseases

In the last decade, many studies have demonstrated that triptolide is a promising neuroprotective agent and alleviates neuroinflammation in animal models of neurodegenerative diseases.

#### Alzheimer’s Disease

Alzheimer’s disease (AD) is a chronic neurodegenerative disease that devastates later decades of life. Increasing evidence suggests that the pathogenesis of AD is not restricted to the neuronal compartment but includes strong interactions with neuroinflammation in the brain (Heneka et al., 2015). The prevalence of AD in worldwide is approximately as high as 24 million. Triptolide could alleviate the immune-inflammatory pathology including Aβ deposition, cytokines expression, and oxidative stress in the treatment of AD.

Triptolide treatment with 0.4 mg/kg/day administration of 18 days could reduce Aβ deposition and neuroinflammation in the hippocampal and cortical areas by upregulating the degradation pathway of Aβ in a transgenic Alzheimer’s disease model (Cheng et al., 2014). Another study found that 20 µg/kg/day triptolide for 8 weeks exerted anti-inflammatory and antioxidative effects on the transgenic mouse brain (Wang et al., 2014). Triptolide inhibited the expression of proinflammatory markers TNFα and IL-1β in the hippocampus in the hippocampus. Recently, Li et al. (2016) demonstrated that 5 µg/kg/day triptolide treatment for 45 days inhibits the activation and proliferation of microglial cells and astrocytes in the hippocampus in a transgenic AD mouse model, reducing neuroinflammation in the brain. Additionally, Cui et al. (2016) found that triptolide with 20 µg/kg/day for 15 weeks alleviates neuroinflammation by suppressing MAPK activity. In the previous studies, APP transgenic mice were used as animal models. The brain of APP transgenic mice is similar to the brain pathology of AD patients with Aβ deposition and neuroinflammation.

Similar to what is observed in animal models of Alzheimer’s disease, the protective effect of triptolide has been found *in vitro*. Xu et al. demonstrated that triptolide has a protective effect in PC12 cells against Aβ_25–35_-induced cytotoxicity by inhibiting the autophagy pathway (Xu et al., 2015). Furthermore, they found that triptolide protects PC12 cells by reducing oxidative stress. Triptolide downregulated the expression of ROS, hydrogen peroxide and MDA induced by Aβ_25–35_ (Xu et al., 2016). PC12 cell line was used to mimic AD *in vitro* model in these studies. Apart from PC12 cell line, human neuroblastoma and human induced pluripotent stem cells are also used as *in vitro* model for AD research. However, these cell lines are not really satisfactory *in vitro* model in order to mimic the features of AD.

#### Parkinson’s Disease

Parkinson’s disease (PD) is a chronic and progressive disorder. Chronic inflammation is a major characteristic of PD (Herrero et al., 2015). There were 6.1 million prevalent cases of PD globally. The worldwide the burden of Parkinson’s disease has more than 6.1 million patients. Triptolide shows anti-inflammatory and neuroprotective effects *in vivo* and *in vitro*. Triptolide protected dopaminergic cells and reduced inflammatory cytokines expression in the brain of PD.

It has been demonstrated that triptolide protects dopaminergic neurons from inflammation-mediated damage. Moreover, 1–10 nM triptolide has been found to suppress LPS-induced activation of microglia and excessive production of TNFα and NO (Li et al., 2004). The administration of triptolide 1 or 5µg/kg for 24 days also attenuated the depletion of dopamine in the striatum and protected dopaminergic neurons from the injury induced by intranigral LPS injection (Zhou et al., 2005). Hu and colleagues have shown that 50 nM triptolide promotes the clearance of pathogenic proteins in neuronal cells using *in vitro* models of PD (Hu et al., 2017).

In addition, using an *in vitro* Parkinson’s disease model of lipopolysaccharides -stimulated PC12 cells, it has been shown that 10–100 ng/ml triptolide downregulates inflammatory mediators such as COX-2 and PGE-2 by inhibiting the NF-κB and MAPK signaling pathways in LPS-stimulated PC12 cells (Geng et al., 2012). Triptolide with concentration more than 200ng/ml showed cytotoxicity effects on PC12 cells. PC12 cells were also used to mimic AD as *in vitro* model. So, this cell lines are not really satisfactory *in vitro* model in order to mimic the features of PD.

#### Multiple Sclerosis

Multiple sclerosis (MS) is a chronic inflammatory autoimmune disease, and experimental autoimmune encephalomyelitis (EAE) is considered an animal model for MS (Constantinescu et al., 2011). MS affects more than 2 million individuals in the worldwide. Triptolide alleviate MS in animal models by reducing inflammation and demyelination pathology.

Kizelsztein et al. (2009) discovered that 100 µg/kg/day triptolide for 2 weeks reduces cellular infiltration and tissue damage in experimental autoimmune encephalomyelitis mice. EAE-promoting cytokines, including IL-2, IL-6 and TNF-α, were reduced in inflamed brains. Molecular analysis revealed that triptolide suppressed NF-κB signaling pathway. Another study conducted by Wang et al. (2008) found that 100 µg/kg/day triptolide for 4 weeks modulates T-cell inflammatory responses and ameliorates EAE. Triptolide inhibited the mRNA expression of both Th1/Th(IL-17) and Th2 cytokines in spleen mononuclear cells and in spinal cord tissues. The levels of IFN-γ, TNF-α, IL-12, IL-6, IL-17 and IL-23 were significantly reduced in both spleen MNCs and spinal cord tissues after triptolide administration. EAE animal model was used to mimic MS in these studies. However, there are some limitations of EAE animal model. Firstly, EAE model offer little information on the progression of MS. EAE is an infectious disorder affecting white matter of spinal cord, whereas MS affects cerebral and cerebellar cortex.

## Conclusion

With an increasing worldwide rate of inflammatory disorders, there is an urgent need for the improvement of the therapeutic activity of anti-inflammatory agents. The anti-inflammatory and immunosuppressive properties of triptolide make it a promising agent to treat inflammatory disorders. In this review, we systemically discussed the potential effects and mechanisms of triptolide in the treatment of different inflammatory diseases including MN, lupus nephritis, kidney transplantation, IBD, asthma, ALI, PAH, etc. Firstly, triptolide could reduce the production of inflammatory mediators such as TNF-α, IL-6, IL-17. Secondly, triptolide alleviated oxidative stress of damaged organs in animal model and related cell lines. Thirdly, triptolide could inhibit the activities of inflammatory cells such as T cells and macrophages. The most related signaling pathway involved in the mechanisms of triptolide was NF-κB and MAPK signaling pathways. Despite the great therapeutic potential of triptolide, there are still some shortcomings in the process of developing it as a new drug. The most studies were focused on the studies of animal models and cell lines. The successful track records of real patients in randomized controlled trials seem very poor. The triptolide cytotoxicity in other healthy organs have not been investigated clearly, either. Hopefully, future stringent preclinical studies on triptolide will provide crucial information regarding its pharmacokinetics and dosage, allowing for further optimization of this compound.

## Author Contributions

KY, XL, QL, QZ, and HJ summarized the literature and drafted the manuscript. KY, XL, TW, GH, and AX revised and edited the manuscript. AX, TW, and GH supervised the work. KY, XL, TW, GH, and AX initiated, finalized, and submitted the manuscript.

## Funding

This work was supported by the National Natural Science Foundation of China (grant numbers 81430099 and 31500704), Projects of International Cooperation and Exchanges (grant number 2014DFA32950) and research program from Beijing University of Chinese Medicine (grant numbers BUCM-2019-JCRC006 and 2019-JYB-TD013).

## Conflict of Interest

The authors declare that the research was conducted in the absence of any commercial or financial relationships that could be construed as a potential conflict of interest.

## References

[B1] Abraham C. Cho J. H. (2009). Inflammatory bowel disease. N. Engl. J. Med. 361, 2066–2078. 10.1056/NEJMra0804647 19923578 PMC3491806

[B2] Bao X. Cui J. Wu Y. Han X. Gao C. Hua Z. (2007). The roles of endogenous reactive oxygen species and nitric oxide in triptolide-induced apoptotic cell death in macrophages. J. Mol. Med. (Berl). 85, 85–98. 10.1007/s00109-006-0113-x 17109129

[B3] Bartok B. Firestein G. S. (2010). Fibroblast-like synoviocytes: key effector cells in rheumatoid arthritis. Immunol. Rev. 233, 233–255. 10.1111/j.0105-2896.2009.00859.x 20193003 PMC2913689

[B4] Bataller R. Brenner D. A. (2005). Liver fibrosis. J. Clin. Invest. 115, 209–218. 10.1172/JCI24282 15690074 PMC546435

[B5] Bell G. M. Anderson A. E. Diboll J. Reece R. Eltherington O. Harry R. A. (2017). Autologous tolerogenic dendritic cells for rheumatoid and inflammatory arthritis. Ann. Rheumatol. Dis. 76, 227–234. 10.1136/annrheumdis-2015-208456 PMC5264217 27117700

[B6] Bustamante M. F. Garcia-Carbonell R. Whisenant K. D. Guma M. (2017). Fibroblast-like synoviocyte metabolism in the pathogenesis of rheumatoid arthritis. Arthritis Res. Ther. 19, 110. 10.1186/s13075-017-1303-3 28569176 PMC5452638

[B7] Cattran D. C. Brenchley P. E. (2017). Membranous nephropathy: integrating basic science into improved clinical management. Kidney Int. 91, 566–574. 10.1016/j.kint.2016.09.048 28065518

[B8] Chan M. A. Kohlmeier J. E. Branden M. Jung M. Benedict S. H. (1999). Triptolide is more effective in preventing T cell proliferation and interferon-gamma production than is FK506. Phytother. Res. 13, 464–467. 10.1002/(sici)1099-1573(199909)13:6<464::aid-ptr483>3.0.co;2-4 10479754

[B9] Chen X. Murakami T. Oppenheim J. J. Howard O. M. (2005). Triptolide, a constituent of immunosuppressive Chinese herbal medicine, is a potent suppressor of dendritic-cell maturation and trafficking. Blood 106, 2409–2416. 10.1182/blood-2005-03-0854 15956285 PMC1569904

[B10] Chen Z. H. Qin W. S. Zeng C. H. Zheng C. X. Hong Y. M. Lu Y. Z. (2010). Triptolide reduces proteinuria in experimental membranous nephropathy and protects against C5b-9-induced podocyte injury *in vitro* . Kidney Int. 77, 974–988. 10.1038/ki.2010.41 20375980

[B11] Chen M. Lv Z. Jiang S. (2011). The effects of triptolide on airway remodelling and transforming growth factor-β_1_/Smad signalling pathway in ovalbumin-sensitized mice. Immunology 132, 376–384. 10.1111/j.1365-2567.2010.03392.x 21214541 PMC3044903

[B12] Chen M. Lv Z. Huang L. Zhang W. Lin X. Shi J. (2015a). Triptolide inhibits TGF-β1-induced cell proliferation in rat airway smooth muscle cells by suppressing Smad signaling. Exp. Cell Res. 331, 362–368. 10.1016/j.yexcr.2014.10.016 25447441

[B13] Chen M. Lv Z. Zhang W. Huang L. Lin X. Shi J. (2015b). Triptolide suppresses airway goblet cell hyperplasia and Muc5ac expression *via* NF-κB in a murine model of asthma. Mol. Immunol. 64, 99–105. 10.1016/j.molimm.2014.11.001 25466609

[B14] Chen C. Yang S. Zhang M. Zhang Z. Hong J. Han D. (2016). Triptolide mitigates radiation-induced pulmonary fibrosis *via* inhibition of axis of alveolar macrophages-NOXes-ROS-myofibroblasts. Cancer Biol. Ther. 17, 381–389. 10.1080/15384047.2016.1139229 27003327 PMC4910907

[B15] Chen Y. Song R. Zhang Y. (2017). Triptolide reduces podocytes injury through blocking ERK and JNK pathways in passive Heymann nephritis (PHN) model. Int. J. Clin. Exp. Med. 10, 692–699.

[B16] Cheng S. LeBlanc K. J. Li L. (2014). Triptolide preserves cognitive function and reduces neuropathology in a mouse model of Alzheimer’s disease. PloS One 9, e108845. 10.1371/journal.pone.0108845 25275487 PMC4183525

[B17] Chong L. W. Hsu Y. C. Chiu Y. T. Yang K. C. Huang Y. T. (2011). Antifibrotic effects of triptolide on hepatic stellate cells and dimethylnitrosamine-intoxicated rats. Phytother. Res. 25, 990–999. 10.1002/ptr.3381 21213358

[B18] Cohn L. Elias J. A. Chupp G. L. (2004). Asthma: mechanisms of disease persistence and progression. Annu. Rev. Immunol. 22, 789–815. 10.1146/annurev.immunol.22.012703.104716 15032597

[B19] Collison J. (2016). Rheumatoid arthritis: Tipping the balance towards resolution. Nat. Rev. Rheumatol. 12, 622. 10.1038/nrrheum.2016.159 27652507

[B20] Constantinescu C. S. Farooqi N. O’Brien K. Gran B. (2011). Experimental autoimmune encephalomyelitis (EAE) as a model for multiple sclerosis (MS). Br. J. Pharmacol. 164, 1079–1106. 10.1111/j.1476-5381.2011.01302.x 21371012 PMC3229753

[B21] Cope A. P. Schulze-Koops H. Aringer M. (2007). The central role of T cells in rheumatoid arthritis. Clin. Exp. Rheumatol. 25, S4–11. 17977483

[B22] Crews G. M. Erickson L. Pan F. Fisniku O. Jang M. S. Wynn C. (2005). Down-regulation of TGF-β and VCAM-1 is associated with successful treatment of chronic rejection in rats. Transplant. Proc. 37, 1926–1928. 10.1016/j.transproceed.2005.02.096 15919506

[B23] Cui Y. Q. Wang Q. Zhang D. M. Wang J. Y. Xiao B. Zheng Y. (2016). Triptolide rescues spatial memory deficits and amyloid-β aggregation accompanied by inhibition of inflammatory responses and MAPKs activity in APP/PS1 transgenic mice. Curr. Alzheimer Res. 13, 288–296. 10.2174/156720501303160217122803 26906357

[B24] Dai S. Yin K. Yao X. Zhou L. (2013). Inhibition of interleukin-13 gene expression by triptolide in activated T lymphocytes. Respirology 18, 1249–1255. 10.1111/resp.12145 23796028

[B25] Davignon J. L. Hayder M. Baron M. Boyer J. F. Constantin A. Apparailly F. (2013). Targeting monocytes/macrophages in the treatment of rheumatoid arthritis. Rheumatol. (Oxford) 52, 590–598. 10.1093/rheumatology/kes304 23204551

[B26] Dong X. G. An Z. M. Guo Y. Zhou J. L. Qin T. (2017). Effect of triptolide on expression of oxidative carbonyl protein in renal cortex of rats with diabetic nephropathy. J. Huazhong Univ. Sci. Technolog. Med. Sci. 37, 25–29. 10.1007/s11596-017-1689-9 28224432

[B27] Farber H. W. Loscalzo J. (2004). Pulmonary arterial hypertension. N. Engl. J. Med. 351, 1655–1665. 10.1056/NEJMra035488 15483284

[B28] Faul J. L. Nishimura T. Berry G. J. Benson G. V. Pearl R. G. Kao P. N. (2000). Triptolide attenuates pulmonary arterial hypertension and neointimal formation in rats. Am. J. Respir. Crit. Care Med. 162, 2252–2258. 10.1164/ajrccm.162.6.2002018 11112148

[B29] Frati G. Schirone L. Chimenti I. Yee D. Biondi-Zoccai G. Volpe M. (2017). An overview of the inflammatory signalling mechanisms in the myocardium underlying the development of diabetic cardiomyopathy. Cardiovasc. Res. 113, 378–388. 10.1093/cvr/cvx011 28395009

[B30] Gao Q. Shen W. Qin W. Zheng C. Zhang M. Zeng C. (2010). Treatment of db/db diabetic mice with triptolide: a novel therapy for diabetic nephropathy. Nephrol. Dial Transplant. 25, 3539–3547. 10.1093/ndt/gfq245 20483955

[B31] Geng Y. Fang M. Wang J. Yu H. Hu Z. Yew D. T. (2012). Triptolide down-regulates COX-2 expression and PGE2 release by suppressing the activity of NF-κB and MAP kinases in lipopolysaccharide-treated PC12 cells. Phytother. Res. 26, 337–343. 10.1002/ptr.3538 21717513

[B32] Goldbach-Mansky R. Wilson M. Fleischmann R. Olsen N. Silverfield J. Kempf P. (2009). Comparison of Tripterygium wilfordii Hook F versus sulfasalazine in the treatment of rheumatoid arthritis: a randomized trial. Ann. Intern. Med. 151, 229–240. 10.7326/0003-4819-151-4-200908180-00005W49-51. 19687490 PMC2938780

[B33] Goldrosen M. H. Straus S. E. (2004). Complementary and alternative medicine: assessing the evidence for immunological benefits. Nat. Rev. Immunol. 4, 912–921. 10.1038/nri1486 15516970

[B34] Gu W. Z. Brandwein S. R. (1998). Inhibition of type II collagen-induced arthritis in rats by triptolide. Int. J. Immunopharmacol. 20, 389–400. 10.1016/s0192-0561(98)00035-6 9778100

[B35] Guo H. Pan C. Chang B. Wu X. Guo J. Zhou Y. (2016). Triptolide improves diabetic nephropathy by regulating Th cell balance and macrophage infiltration in rat models of diabetic nephropathy. Exp. Clin. Endocrinol. Diabetes 124, 389–398. 10.1055/s-0042-106083 27328403

[B36] Guo X. Xue M. Li C. J. Yang W. Wang S. S. Ma Z. J. (2016). Protective effects of triptolide on TLR4 mediated autoimmune and inflammatory response induced myocardial fibrosis in diabetic cardiomyopathy. J. Ethnopharmacol. 193, 333–344. 10.1016/j.jep.2016.08.029 27558948

[B37] Han F. Xue M. Chang Y. Li X. Yang Y. Sun B. (2017). Triptolide suppresses glomerular mesangial cell proliferation in diabetic nephropathy is associated with inhibition of PDK1/Akt/mTOR pathway. Int. J. Biol. Sci. 13, 1266–1275. 10.7150/ijbs.20485 29104493 PMC5666525

[B38] Heneka M. T. Carson M. J. El Khoury J. Landreth G. E. Brosseron F. Feinstein D. L. (2015). Neuroinflammation in Alzheimer’s disease. Lancet Neurol. 14, 388–405. 10.1016/S1474-4422(15)70016-5 25792098 PMC5909703

[B39] Herrero M. T. Estrada C. Maatouk L. Vyas S. (2015). Inflammation in Parkinson’s disease: role of glucocorticoids. Front. Neuroanat. 9, 32. 10.3389/fnana.2015.00032 25883554 PMC4382972

[B40] Ho L. J. Chang W. L. Chen A. Chao P. Lai J. H. (2013). Differential immunomodulatory effects by Tripterygium wilfordii Hook f-derived refined extract PG27 and its purified component PG490 (triptolide) in human peripheral blood T cells: potential therapeutics for arthritis and possible mechanisms explaining in part Chinese herbal theoryJunn-Chenn-Zuou-SS. J. Transl. Med. 11, 294. 10.1186/1479-5876-11-294 24256769 PMC4222270

[B41] Hong Y. Zhou W. Li K. Sacks S. H. (2002). Triptolide is a potent suppressant of C3, CD40 and B7h expression in activated human proximal tubular epithelial cells. Kidney Int. 62, 1291–1300. 10.1111/j.1523-1755.2002.kid586.x 12234299

[B42] Hoyle G. W. Hoyle C. I. Chen J. Chang W. Williams R. W. Rando R. J. (2010). Identification of triptolide, a natural diterpenoid compound, as an inhibitor of lung inflammation. Am. J. Physiol. Lung Cell Mol. Physiol. 298, L830–L836. 10.1152/ajplung.00014.2010 20348278 PMC2886612

[B43] Hu G. Gong X. Wang L. Liu M. Liu Y. Fu X. (2017). Triptolide promotes the clearance of α-synuclein by enhancing autophagy in neuronal cells. Mol. Neurobiol. 54, 2361–2372. 10.1007/s12035-016-9808-3 26957304

[B44] Huang J. Zhou L. Wu H. Pavlos N. Chim S. M. Liu Q. (2015). Triptolide inhibits osteoclast formation, bone resorption, RANKL-mediated NF-κB activation and titanium particle-induced osteolysis in a mouse model. Mol. Cell Endocrinol. 399, 346–353. 10.1016/j.mce.2014.10.016 25448849

[B45] Ingulli E. (2010). Mechanism of cellular rejection in transplantation. Pediatr. Nephrol. 25, 61–74. 10.1007/s00467-008-1020-x 21476231 PMC2778785

[B46] Ji N. F. Wang W. J. Zhang M. S. Huang M. (2015). Triptolide modulates the CD4^+^ T cells balance in an allergic asthmatic mouse model. Eur. Respir. J. 46, PA4371. 10.1183/13993003.congress-2015.PA4371

[B47] Khan S. Greenberg J. D. Bhardwaj N. (2009). Dendritic cells as targets for therapy in rheumatoid arthritis. Nat. Rev. Rheumatol. 5, 566–571. 10.1038/nrrheum.2009.185 19798032 PMC2884969

[B48] Kim Y. H. Lee S. H. Lee J. Y. Choi S. W. Park J. W. Kwon T. K. (2004). Triptolide inhibits murine-inducible nitric oxide synthase expression by down-regulating lipopolysaccharide-induced activity of nuclear factor-κB and c-Jun NH2-terminal kinase. Eur. J. Pharmacol. 494, 1–9. 10.1016/j.ejphar.2004.04.040 15194445

[B49] Kinne R. W. Stuhlmüller B. Burmester G. R. (2007). Cells of the synovium in rheumatoid arthritis. Macrophages. Arthritis Res. Ther. 9, 224. 10.1186/ar2333 18177511 PMC2246244

[B50] Kizelsztein P. Komarnytsky S. Raskin I. (2009). Oral administration of triptolide ameliorates the clinical signs of experimental autoimmune encephalomyelitis (EAE) by induction of HSP70 and stabilization of NF-κB/IκBα transcriptional complex. J. Neuroimmunol. 217, 28–37. 10.1016/j.jneuroim.2009.08.017 19796825

[B51] Kupchan S. M. Court W. A. Dailey R. G. Jr. Gilmore C. J. Bryan R. F. (1972). Triptolide and tripdiolide: novel antileukemic diterpenoid triepoxides from Tripterygium wilfordii. J. Am. Chem. Soc 94, 7194–7195. 10.1021/ja00775a078 5072337

[B52] Kusunoki N. Yamazaki R. Kitasato H. Beppu M. Aoki H. Kawai S. (2004). Triptolide, an active compound identified in a traditional Chinese herb, induces apoptosis of rheumatoid synovial fibroblasts. BMC Pharmacol. 4, 2. 10.1186/1471-2210-4-2 15040811 PMC373449

[B53] Lech M. Anders H. J. (2013). The pathogenesis of lupus nephritis. J. Am. Soc Nephrol. 24, 1357–1366. 10.1681/ASN.2013010026 23929771 PMC3752952

[B54] Li F. Q. Lu X. Z. Liang X. B. Zhou H. F. Xue B. Liu X. Y. (2004). Triptolide, a Chinese herbal extract, protects dopaminergic neurons from inflammation-mediated damage through inhibition of microglial activation. J. Neuroimmunol. 148, 24–31. 10.1016/j.jneuroim.2003.10.054 14975583

[B55] Li Y. Yu C. Zhu W. M. Xie Y. Qi X. Li N. (2010). Triptolide ameliorates IL-10-deficient mice colitis by mechanisms involving suppression of IL-6/STAT3 signaling pathway and down-regulation of IL-17. Mol. Immunol. 47, 2467–2474. 10.1016/j.molimm.2010.06.007 20615550

[B56] Li J. M. Zhang Y. Tang L. Chen Y. H. Gao Q. Bao M. H. (2016). Effects of triptolide on hippocampal microglial cells and astrocytes in the APP/PS1 double transgenic mouse model of Alzheimer’s disease. Neural. Regen. Res. 11, 1492–1498. 10.4103/1673-5374.191224 27857756 PMC5090855

[B57] Liacini A. Sylvester J. Zafarullah M. (2005). Triptolide suppresses proinflammatory cytokine-induced matrix metalloproteinase and aggrecanase-1 gene expression in chondrocytes. Biochem. Biophys. Res. Commun. 327, 320–327. 10.1016/j.bbrc.2004.12.020 15629465

[B58] Liang Z. Leo S. Wen H. Ouyang M. Jiang W. Yang K. (2015). Triptolide improves systolic function and myocardial energy metabolism of diabetic cardiomyopathy in streptozotocin-induced diabetic rats. BMC Cardiovasc. Disord. 15, 42. 10.1186/s12872-015-0030-4 25967112 PMC4431461

[B59] Lin N. Sato T. Ito A. (2001). Triptolide, a novel diterpenoid triepoxide from Tripterygium wilfordii Hook. f., suppresses the production and gene expression of pro-matrix metalloproteinases 1 and 3 and augments those of tissue inhibitors of metalloproteinases 1 and 2 in human synovial fibroblasts. Arthritis Rheumatol. 44, 2193–2200. 10.1002/1529-0131(200109)44:9<2193:aid-art373>3.0.co;2-5 11592385

[B60] Lin N. Liu C. Xiao C. Jia H. Imada K. Wu H. (2007). Triptolide, a diterpenoid triepoxide, suppresses inflammation and cartilage destruction in collagen-induced arthritis mice. Biochem. Pharmacol. 73, 136–146. 10.1016/j.bcp.2006.08.027 17097618

[B61] Liu H. Liu Z. H. Chen Z. H. Yang J. W. Li L. S. (2000). Triptolide: a potent inhibitor of NF-κB in T-lymphocytes. Acta Pharmacol. Sin. 21, 782–786. 11501157

[B62] Liu Q. Chen T. Chen H. Zhang M. Li N. Lu Z. (2004). Triptolide (PG-490) induces apoptosis of dendritic cells through sequential p38 MAP kinase phosphorylation and caspase 3 activation. Biochem. Biophys. Res. Commun. 319, 980–986. 10.1016/j.bbrc.2004.04.201 15184078

[B63] Liu J. Wu Q. L. Feng Y. H. Wang Y. F. Li X. Y. Zuo J. P. (2005). Triptolide suppresses CD80 and CD86 expressions and IL-12 production in THP-1 cells. Acta Pharmacol. Sin. 26, 223–227. 10.1111/j.1745-7254.2005.00035.x 15663903

[B64] Liu Q. Chen T. Chen G. Li N. Wang J. Ma P. (2006). Immunosuppressant triptolide inhibits dendritic cell-mediated chemoattraction of neutrophils and T cells through inhibiting Stat3 phosphorylation and NF-κB activation. Biochem. Biophys. Res. Commun. 345, 1122–1130. 10.1016/j.bbrc.2006.05.024 16713992

[B65] Liu Q. Chen T. Chen G. Shu X. Sun A. Ma P. (2007). Triptolide impairs dendritic cell migration by inhibiting CCR7 and COX-2 expression through PI3-K/Akt and NF-κB pathways. Mol. Immunol. 44, 2686–2696. 10.1016/j.molimm.2006.12.003 17223196

[B66] Liu C. Zhang Y. Kong X. Zhu L. Pang J. Xu Y. (2013). Triptolide prevents bone destruction in the collagen-induced arthritis model of rheumatoid arthritis by targeting RANKL/RANK/OPG signal pathway. Evid. Based Complement Alternat. Med. 2013, 626038. 10.1155/2013/626038 23573139 PMC3610373

[B67] Lu Y. Wang W. J. Leng J. H. Cheng L. F. Feng L. Yao H. P. (2008). Inhibitory effect of triptolide on interleukin-18 and its receptor in rheumatoid arthritis synovial fibroblasts. Inflamm. Res. 57, 260–265. 10.1007/s00011-007-7128-9 18516710

[B68] Lv Q. W. Zhang W. Shi Q. Zheng W. J. Li X. Chen H. (2015). Comparison of Tripterygium wilfordii Hook F with methotrexate in the treatment of active rheumatoid arthritis (TRIFRA): a randomised, controlled clinical trial. Ann. Rheumatol. Dis. 74, 1078–1086. 10.1136/annrheumdis-2013-204807 24733191

[B69] Ma R. Liu L. Liu X. Wang Y. Jiang W. Xu L. (2013). Triptolide markedly attenuates albuminuria and podocyte injury in an animal model of diabetic nephropathy. Exp. Ther. Med. 6, 649–656. 10.3892/etm.2013.1226 24137241 PMC3786875

[B70] Mao H. Chen X. R. Yi Q. Li S. Y. Wang Z. L. Li F. Y. (2008). Mycophenolate mofetil and triptolide alleviating airway inflammation in asthmatic model mice partly by inhibiting bone marrow eosinophilopoiesis. Int. Immunopharmacol. 8, 1039–1048. 10.1016/j.intimp.2008.03.009 18486916

[B71] Matta R. Wang X. Ge H. Ray W. Nelin L. D. Liu Y. (2009). Triptolide induces anti-inflammatory cellular responses. Am. J. Transl. Res. 1, 267–282. 19956437 PMC2776323

[B72] Navarro-González J. F. Mora-Fernández C. (2008). The role of inflammatory cytokines in diabetic nephropathy. J. Am. Soc. Nephrol. 19, 433–442. 10.1681/ASN.2007091048 18256353

[B73] Otero M. Goldring M. B. (2007). Cells of the synovium in rheumatoid arthritis. Chondrocytes. Arthritis Res. Ther. 9, 220. 10.1186/ar2292 18001488 PMC2212563

[B74] Park S. W. Kim Y. I. (2013). Triptolide induces apoptosis of PMA-treated THP-1 cells through activation of caspases, inhibition of NF-κB and activation of MAPKs. Int. J. Oncol. 43, 1169–1175. 10.3892/ijo.2013.2033 23900299

[B75] Premkumar V. Dey M. Dorn R. Raskin I. (2010). MyD88-dependent and independent pathways of Toll-Like Receptors are engaged in biological activity of Triptolide in ligand-stimulated macrophages. BMC Chem. Biol. 10, 3. 10.1186/1472-6769-10-3 20385024 PMC2873377

[B76] Qiu D. Zhao G. Aoki Y. Shi L. Uyei A. Nazarian S. (1999). Immunosuppressant PG490 (triptolide) inhibits T-cell interleukin-2 expression at the level of purine-box/nuclear factor of activated T-cells and NF-κB transcriptional activation. J. Biol. Chem. 274, 13443–13450. 10.1074/jbc.274.19.13443 10224109

[B77] Rieder F. Fiocchi C. (2008). Intestinal fibrosis in inflammatory bowel disease - Current knowledge and future perspectives. J. Crohns Colitis 2, 279–290. 10.1016/j.crohns.2008.05.009 21172225

[B78] Rubenfeld G. D. Caldwell E. Peabody E. Weaver J. Martin D. P. Neff M. (2005). Incidence and outcomes of acute lung injury. N. Engl. J. Med. 353, 1685–1693. 10.1056/NEJMoa050333 16236739

[B79] Tao X. Davis L. S. Lipsky P. E. (1991). Effect of an extract of the Chinese herbal remedy Tripterygium wilfordii Hook F on human immune responsiveness. Arthritis Rheumatol. 34, 1274–1281. 10.1002/art.1780341011 1930317

[B80] Tao X. Fan F. Hoffmann V. Gao C. Y. Longo N. S. Zerfas P. (2008). Effective therapy for nephritis in (NZB x NZW)F1 mice with triptolide and tripdiolide, the principal active components of the Chinese herbal remedy Tripterygium wilfordii Hook F. Arthritis Rheumatol. 58, 1774–1783. 10.1002/art.23513 PMC3560846 18512813

[B81] Tao Q. Wang B. Zheng Y. Li G. Ren J. (2015). Triptolide ameliorates colonic fibrosis in an experimental rat model. Mol. Med. Rep. 12, 1891–1897. 10.3892/mmr.2015.3582 25845760 PMC4464197

[B82] Vaszar L. T. Nishimura T. Storey J. D. Zhao G. Qiu D. Faul J. L. (2004). Longitudinal transcriptional analysis of developing neointimal vascular occlusion and pulmonary hypertension in rats. Physiol. Genomics 17, 150–156. 10.1152/physiolgenomics.00198.2003 15082832

[B83] Wang B. Ma L. Tao X. Lipsky P. E. (2004). Triptolide, an active component of the Chinese herbal remedy Tripterygium wilfordii Hook F, inhibits production of nitric oxide by decreasing inducible nitric oxide synthase gene transcription. Arthritis Rheumatol. 50, 2995–2303. 10.1002/art.20459 15457469

[B84] Wang Y. Mei Y. Feng D. Xu L. (2008). Triptolide modulates T-cell inflammatory responses and ameliorates experimental autoimmune encephalomyelitis. J. Neurosci. Res. 86, 2441–2449. 10.1002/jnr.21683 18438925

[B85] Wang Z. Jin H. Xu R. Mei Q. Fan D. (2009). Triptolide downregulates Rac1 and the JAK/STAT3 pathway and inhibits colitis-related colon cancer progression. Exp. Mol. Med. 41, 717–727. 10.3858/emm.2009.41.10.078 19561401 PMC2772974

[B86] Wang Q. Xiao B. Cui S. Song H. Qian Y. Dong L. (2014). Triptolide treatment reduces Alzheimer’s disease (AD)-like pathology through inhibition of BACE1 in a transgenic mouse model of AD. Dis. Model Mech. 7, 1385–1395. 10.1242/dmm.018218 25481013 PMC4257007

[B87] Wang X. Zhang L. Duan W. Liu B. Gong P. Ding Y. (2014). Anti-inflammatory effects of triptolide by inhibiting the NF-κB signalling pathway in LPS-induced acute lung injury in a murine model. Mol. Med. Rep. 10, 447–452. 10.3892/mmr.2014.2191 24789089

[B88] Wei D. Huang Z. (2014). Anti-inflammatory effects of triptolide in LPS-induced acute lung injury in mice. Inflammation 37, 1307–1316. 10.1007/s10753-014-9858-5 24706025

[B89] Wei X. Gong J. Zhu J. Wang P. Li N. Zhu W. (2008). The suppressive effect of triptolide on chronic colitis and TNF-α/TNFR2 signal pathway in interleukin-10 deficient mice. Clin. Immunol. 129, 211–218. 10.1016/j.clim.2008.07.018 18757245

[B90] Wen H. L. Liang Z. S. Zhang R. Yang K. (2013). Anti-inflammatory effects of triptolide improve left ventricular function in a rat model of diabetic cardiomyopathy. Cardiovasc. Diabetol. 12, 50. 10.1186/1475-2840-12-50 23530831 PMC3617021

[B91] Wu Y. Cui J. Bao X. Chan S. Young D. O. Liu D. (2006). Triptolide attenuates oxidative stress, NF-κB activation and multiple cytokine gene expression in murine peritoneal macrophage. Int. J. Mol. Med. 17, 141–150. 16328023

[B92] Xiao C. Zhou J. He Y. Jia H. Zhao L. Zhao N. (2009). Effects of triptolide from Radix Tripterygium wilfordii (Leigongteng) on cartilage cytokines and transcription factor NF-κB: a study on induced arthritis in rats. Chin. Med. 4, 13. 10.1186/1749-8546-4-13 19570240 PMC2709898

[B93] Xu P. Li Z. Wang H. Zhang X. Yang Z. (2015). Triptolide inhibited cytotoxicity of differentiated PC12 cells induced by Amyloid-Beta_25-35_ *via the* autophagy pathway. PloS One 10, e0142719. 10.1371/journal.pone.0142719 26554937 PMC4640509

[B94] Xu H. Zhao H. Lu C. Qiu Q. Wang G. Huang J. (2016). Triptolide inhibits osteoclast differentiation and bone resorption *in vitro via* enhancing the production of IL-10 and TGF-β1 by regulatory T cells. Mediators Inflammation, 2016, 8048170. 10.1155/2016/80481702016 PMC4930824 27413257

[B95] Xu P. Wang H. Li Z. Yang Z. (2016). Triptolide attenuated injury *via* inhibiting oxidative stress in Amyloid-Beta_25-35_-treated differentiated PC12 cells. Life Sci. 145, 19–26. 10.1016/j.lfs.2015.12.018 26679104

[B96] Yang Y. Liu Z. Tolosa E. Yang J. Li L. (1998). Triptolide induces apoptotic death of T lymphocyte. Immunopharmacology 40, 139–149. 10.1016/s0162-3109(98)00036-8 9826028

[B97] Yang F. Bai X. J. Hu D. Li Z. F. Liu K. J. (2010). Effect of triptolide on secretion of inflammatory cellular factors TNF-α and IL-8 in peritoneal macrophages of mice activated by lipopolysaccharide. World J. Emerg. Med. 1, 70–74. 25214945 PMC4129760

[B98] Yang S. Zhang M. Chen C. Cao Y. Tian Y. Guo Y. (2015). Triptolide mitigates radiation-induced pulmonary fibrosis. Radiat. Res. 184, 509–517. 10.1667/RR13831.1 26488756

[B99] Yang Y. Ye Y. Qiu Q. Xiao Y. Huang M. Shi M. (2016). Triptolide inhibits the migration and invasion of rheumatoid fibroblast-like synoviocytes by blocking the activation of the JNK MAPK pathway. Int. Immunopharmacol. 41, 8–16. 10.1016/j.intimp.2016.10.005 27816728

[B100] Yuan X. P. He X. S. Wang C. X. Liu L. S. Fu Q. (2011). Triptolide attenuates renal interstitial fibrosis in rats with unilateral ureteral obstruction. Nephrol. (Carlton) 16, 200–210. 10.1111/j.1440-1797.2010.01359.x 21272133

[B101] Zhang G. Liu Y. Guo H. Sun Z. Zhou Y. H. (2009). Triptolide promotes generation of FoxP3+ T regulatory cells in rats. J. Ethnopharmacol. 125, 41–46. 10.1016/j.jep.2009.06.020 19560530

[B102] Zhang G. Chen J. Liu Y. Yang R. Guo H. Sun Z. (2013). Triptolide-conditioned dendritic cells induce allospecific T-cell regulation and prolong renal graft survival. J. Invest. Surg. 26, 191–199. 10.3109/08941939.2012.737408 23514053

[B103] Zhang H. Zhang X. Ding X. Cao W. Qu L. Zhou G. (2014). Effect of secondary lymphoid tissue chemokine suppression on experimental ulcerative colitis in mice. Genet. Mol. Res. 13, 3337–3345. 10.4238/2014.April.29.12 24841666

[B104] Zhang H. Gong C. Qu L. Ding X. Cao W. Chen H. (2016). Therapeutic effects of triptolide via the inhibition of IL-1β expression in a mouse model of ulcerative colitis. Exp. Ther. Med. 12, 1279–1286. 10.3892/etm.2016.3490 27588050 PMC4997980

[B105] Zhou D. Liu Y. (2016). Renal fibrosis in 2015: Understanding the mechanisms of kidney fibrosis. Nat. Rev. Nephrol. 12, 68–70. 10.1038/nrneph.2015.215 26714578 PMC4868356

[B106] Zhou H. F. Liu X. Y. Niu D. B. Li F. Q. He Q. H. Wang X. M. (2005). Triptolide protects dopaminergic neurons from inflammation-mediated damage induced by lipopolysaccharide intranigral injection. Neurobiol. Dis. 18, 441–449. 10.1016/j.nbd.2004.12.005 15755670

[B107] Zhou Y. Hong Y. Huang H. (2016). Triptolide attenuates inflammatory response in membranous glomerulo-nephritis rat *via* downregulation of NF-κB signaling pathway. Kidney Blood Press Res. 41, 901–910. 10.1159/000452591 27871079

[B108] Zhou Y. Y. Xia X. Peng W. K. Wang Q. H. Peng J. H. Li Y. L. (2018). The Effectiveness and Safety of Tripterygium wilfordii Hook. F Extracts in Rheumatoid Arthritis: A Systematic Review and Meta-Analysis. Front. Pharmacol. 9, 356. 10.3389/fphar.2018.00356 29713281 PMC5911475

[B109] Zhu K. J. Shen Q. Y. Cheng H. Mao X. H. Lao L. M. Hao G. L. (2005). Triptolide affects the differentiation, maturation and function of human dendritic cells. Int. Immunopharmacol. 5, 1415–1426. 10.1016/j.intimp.2005.03.020 15953568

